# Gene Targets Network Analysis for the Revealing and Guidance of Molecular Driving Mechanism of Lung Cancer

**DOI:** 10.3389/fgene.2021.727201

**Published:** 2021-09-20

**Authors:** Risheng Huang, Xiao Xiang, Kangliang Zhang, Yuanliang Zheng, Chichao Wang, Guanqiong Hu

**Affiliations:** ^1^Department of Thoracic Surgery, Dingli Clinical College of Wenzhou Medical University (Wenzhou Central Hospital), Wenzhou, China; ^2^Department of Breast Surgery, First Affiliated Hospital of Wenzhou Medical University, Wenzhou, China; ^3^Central Laboratory, Dingli Clinical College of Wenzhou Medical University (Wenzhou Central Hospital), Wenzhou, China; ^4^Department of Pediatric, First Affiliated Hospital of Wenzhou Medical University, Wenzhou, China

**Keywords:** gene target network analysis, lung cancer, molecular driving mechanism, nursing guidance, protein interaction

## Abstract

The objective was to explore the function of gene differential expressions between lung cancer tissues and the interaction between the relevant encoded proteins, thereby analyzing the important genes closely related to lung cancer. A total of 120 samples from the GEO database (including two groups, i.e., 60 lung cancer *in situ* specimens and 60 normal specimens) were taken as the research objects, which were submitted to the analysis of signaling pathway, biological function enrichment, and protein interactions to reveal the molecular driving mechanism of lung cancer. Results: A total of 875 differentially expressed genes were obtained, including 291 up-regulated genes and 584 down-regulated genes. The up-regulated genes were mainly involved in biological processes such as protein metabolism, protein hydrolysis, mitosis, and cell division. Down-regulated genes were mainly involved in neutrophil chemotaxis, inflammatory response, immune response, and angiogenesis. The protein expression of high expression genes and low expression genes in patients were higher than those in the control group. The protein corresponding to the high expression gene was highly expressed in the patient group. Meanwhile, the proteins corresponding to the low expression genes were also expressed in the patient group, which showed that although the proteins corresponding to the low expression genes were low in the patients, they were still the target genes related to lung cancer. In conclusion, the molecular driving mechanism in lung cancer was mainly related to protein metabolism, proteolysis, mitosis, and cell division. It was found that TOP2A, CCNB1, CCNA2, CDK1, and TTK might be the critical target genes of lung cancer.

## Introduction

The incidence rate of lung cancer is one of the fastest growing malignant tumors (Masters et al., 2017). Lung cancer is mainly divided into small cell lung cancer (SCLC) and non-small cell lung cancer (NSCLC), of which NSCLC accounts for 80% of all lung cancer cases (Shi et al., 2017). Currently, molecular-targeted drug therapy takes the molecules that block the high expression of cancer cell membrane or cells as the therapeutic target, reduces the fragmentation effect on normal cells by blocking the growth, infiltration, metastasis, and inducing apoptosis of normal cells, and reduces the incidence of adverse drug reactions in patients (Shen et al., 2018; Xu et al., 2018). In the absence of biopsy, the blood samples of patients with lung cancer are the only source of information for analyzing clinically relevant genetic changes, including epidermal growth factor receptor (EGFR), Kirsten rat sarcoma viral oncogene (KRAS), v-raf murine sarcoma viral oncogene homolog B1 (BRAF), c-ros oncogene 1 (ROS1), and anaplastic lymphoma kinase (ALK) (Allan-Blitz et al., 2018). As new treatment options emerge, predictive detection of lung cancer has become a research hot spot in medical field (Horimasu et al., 2017). The diagnosis of lung cancer diseases mainly includes the identification and classification of malignant tumors, molecular tests, and immunohistochemical analysis. Complex diagnostic analysis algorithms have evolved, requiring specific drugs tailored to individual patients and considering the way to make investigations and diagnostic strategies based on individual tumors (Zhang et al., 2018). Some studies have reported that KRAS mutations may be the targets for preventing and treating KRAS mutant lung cancer and other tumor diseases (Krasnov et al., 2017). Studies have shown that the molecular driving mechanisms of lung cancer in different tumor stages are also different, and NKTR may be the target of prevention and treatment of lung cancer diseases (Zhou et al., 2017). Some studies have used the CIBERSORT method to identify and quantify the number of different cells in a tumor sample by reference genes combined with machine learning. Such an approach solves one of the major problems in determining cell types to some extent by using the reference genes (Zins et al., 2018). CIBERSORT is used to estimate the abundance of member cell types in mixed cell population by using gene expression data. It is a tool of bioinformatics analysis method and has important application value in the field of molecular biology.

Bioinformatics uses computers to mine and analyze great information in biological databases, focuses on gene and proteomic analysis, and is widely used in the fields of molecular genetics and genomics. In the field of tumor research, bioinformatics combines suspicious tumor genes with known biological data through the biological network analysis of tumor-related pathways and biological processes, identifies tumor-related functional categories, and excavates tumor networks. It also predicts potential pathogenic proteins and plays an important role in tumor pathogenesis, diagnosis, and treatment. As the gene chip technology continuously develops, it has become a hot topic how to process and analyze tremendous data and find more effective information. At present, gene chip technology is mainly used in the research of tumor-related gene information, such as screening tumor-related genes, measuring tumor mutation genes, studying tumor gene expression profiles, and diagnosing tumor diseases. In this way, it can explore the extent of influences of genetic, environmental, and pharmaceutical factors for tumors on the expression of related genes during the occurrence and development of tumors.

The rapid development of high-throughput technologies, such as MeDip-seq, methylated microarrays, and RNA-seq, has provided technical support for the identification of biomarkers for a variety of diseases such as lung cancer, as well as opportunities for the availability of publicly available data sets. By selecting the gene expression dataset of lung cancer, this study innovatively explores the network of lung cancer target genes through gene expression analysis of different databases, thus exploring the molecular driving mechanism of lung cancer and providing reference for clinical molecular drug treatment and nursing guidance of lung cancer.

## Materials and Methods

### Data Resource and Processing

A total of 120 samples of lung cancer mRNA sample GSE19408 (including two groups: 60 lung cancer *in situ* specimens and 60 normal specimens) were selected from the GEO (Gene Expression Omnibus) database, using open-source software R3.4.2. for preprocessing the differential analysis of sample data.

First, download the sample, import the CEL (cool edit loop) format file into the R program, use the limma package in the R language to count the difference between the lung cancer gene and the normal gene, and then follow the FDR (false discovery rate) and FC (fold change, gene expression fold ratio) from which differentially expressed genes were selected, and the comparison between the two groups of genes must satisfy the requirements of FDR < 0.01 and | log2 FC | ≥ 1.

### Signal Pathway Analysis and Biological Function Enrichment

Signaling pathway analysis and biological function enrichment of the screened NSCLC differentially expressed genes were performed using the Functional annotation chart tool under the DAVID platform. First, the differentially expressed genes were introduced into the DAVID list in the form of gene symbol, and the humans were submitted to the task in the species type, and the GO (Gene Ontology) analysis and the KEGG (Kyoto Encyclopedia of Genes and Genomes) pathway were performed on both the up-regulated and down-regulated genes (Wang et al., 2020). After the results were obtained, the differentially expressed genes with statistical significance (*P* ≤ 0.01) were selected.

### Protein Interaction Analysis

Gene data can be applied to gene regulatory network analysis to analyze the differential expression of genes for studying the differential expression of their target genes and the processes that constitute various organisms, such as organ formation, embryo development, and disease pathogenesis. The network of relationships is compared between cell types or states and analyzed further, and specific molecular features and functional blocks can be identified, which are the basis for state transitions. In order to identify key target genes related to lung cancer, this study established a protein interaction network model to explore the regulatory relationship of differential genes at the protein level. The differentially expressed genes obtained by the DAVID platform were subjected to ID (Identity Document) conversion and input into the STRING 9.1 (the Search Tool for the Retrieval of Interacting Genes) database to establish a differentially expressed gene encoding protein-protein between Interaction network diagram. Proteins at the center of the protein-protein interaction network often play a relatively important role in the development of the disease. The selection criteria for PPI (Protein-protein interaction network) analysis was combination score >0.4 (medium confidence). Enter the PPI value into the visualization tool, that is, the Cytoscape software, and use the analysis plug-in to calculate the edge of the nodes in the network to get the number of protein interactions (Degree). The analysis steps of Cytoscape software are as follows: first, import the node attribute file, file- >import- >table- >file(node.txt) (here is table instead of network), and then set the format of simple network diagram in style. Finally, export the file. The data can be network file, table file, or picture file. The picture file includes a variety of picture formats and PDF format, which can be selected in the toolbar.

### Western Blotting Detection

(1) Total protein extraction: Cells were taken out; the culture medium was discarded, and the cells were washed with PBS. Then, 70 μL of cell lysate was added to each well. After 5 min, the cell suspension was transferred to an Eppendorf (EP) tube (TIANGEN Biochemical Technology (Beijing) Co., Ltd., China) and shaken once every 5 min for a total of 6 times. The cell suspension was put into a 4°C centrifuge, centrifuged at 1,000 rpm/min for 15 min. The supernatant was taken for bicinchoninic acid (BCA) protein quantitative determination, and the standard curve was drawn.
(2) Preparation of stacking gel and separation gel: The reagents (purchased from TIANGEN Biochemical Technology (Beijing) Co., Ltd., China) were summarized in Table 1 below:

**TABLE 1:** Configuration of stacking gel and separation gel.

Ingredients	Stacking gel	10% Separation gel
Double distilled water	2.6 × 10^3^	3 × 10^3^
30% polyacrylamide liquid	0.64 × 10^3^	3 × 10^3^
1.0 mol/L tromethamine (Tris, pH8.8)	2.3 × 10^3^	2.3 × 10^3^
10% sodium dodecyl sulfate (SDS)	0.03 × 10^3^	0.1 × 10^3^
10% ammonium persulfate	0.04 × 10^3^	0.1 × 10^3^
Tetramethylethylenediamine (TEMED)	0.004 × 10^3^	0.004 × 10^3^

(3) Electrophoresis and image development: The glass plate was cleaned thoroughly with distilled water and ethanol. The glass plate was aligned and put in the clamp vertically on the glue rack. The distilled water was added to the glass plate to a suitable position. Then, the device was stood for 8 min to test whether the glass plate was leaking. A 10% separation gel was prepared according to the formula in Table 1. After mixing, 6 mL was added to the gap in the middle of the glass plate with a pipette; then, 3 mL of isopropanol was added slowly. Under 37°C condition, once a refraction line appeared between isopropanol and the separation gel, the separation gel solidified. Afterward, the isopropanol was poured out, and the device was washed with distilled water three times for later use. After the stacking gel was configured, 3 mL was added to the glass plate, which should slowly enter the comb to prevent bubbles.

After the concentrated gel was solidified, the glass plate and the plastic replacement plate were sandwiched in the rack with electrodes; then, the device was put into the electrophoresis tank, and the comb was pulled out. Next, 30 μL of the expressed protein supernatant was taken out, added with 10 μL of 5 × loading buffer, mixed evenly, and boiled for 10 min at 100°C.

Eventually, 40 μL of the sample was loaded on each well of the electrophoresis gel. Under 80V voltage, the bromophenol blue formed a straight line in the gel, and then the voltage was changed to 120V. When the bromophenol blue ran to the lower edge, the power supply was disconnected, and the membrane was transferred. The membrane transfer process is as follows: soak the glue in the transfer buffer for 10 min, cut six pieces of membrane and filter paper according to the size of the glue, put the transfer buffer for 10 min, place each layer in the order of sponge/3 layers of filter paper/glue/membrane/3 layers of filter paper/sponge, and drive away the bubbles with a test tube. Then put the transfer tank into the ice bath, put the above interlayer, add transfer buffer, and insert the electrode, 100V for 1 h.

After the membrane transfer was completed, the gel image processing system (Unverbindlicher Verkaufspreis, Germany) was used to analyze the target band’s molecular weight and net optical density. The relative expression of target protein = target band gray value OD/internal reference gray value OD.

## Results and Discussion

### Influence of Patients’ Clinical Characteristics on Their Quality of Life

**FIGURE 1:**
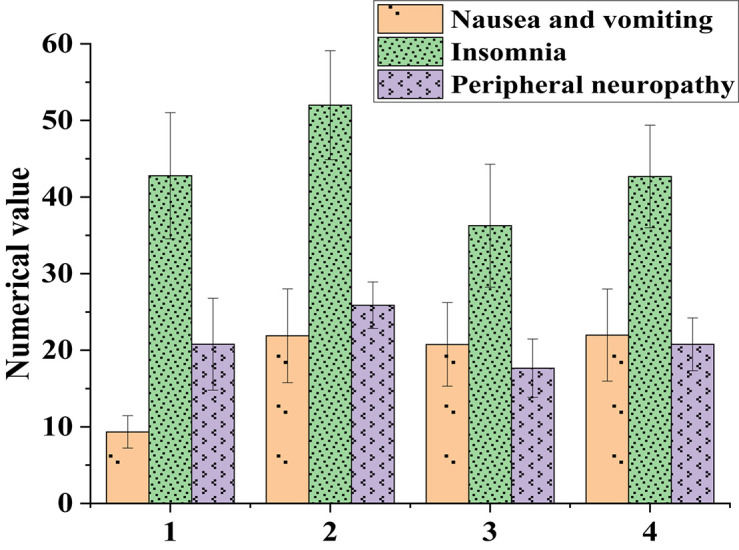
Influences of patients’ clinical characteristics on their quality of life (1: Stage I-II; 2: Stage III-IV; 3: Chemotherapy less than 3 times; 4: Chemotherapy more than 3 times).

Figure 1 presented the basic clinical characteristics of 60 patients.

Figure 1 suggested that patients in stage III-IV had more severe symptoms, including nausea, vomiting, insomnia, and peripheral neuropathy, than patients in stage I-II. Patients who received more than three chemotherapies had more severe nausea, vomiting, insomnia, and peripheral neuropathy than those who received less than three chemotherapies. This indicated that the more times the chemotherapy patients had, the greater the side effects of the body were. How to make cancer patients achieve the best therapeutic effect within the minimum number of chemotherapy is not only a difficult problem of anti-cancer treatment, but also a key research direction.

### Lung Cancer Differential Expression Gene Analysis Results

**FIGURE 2:**
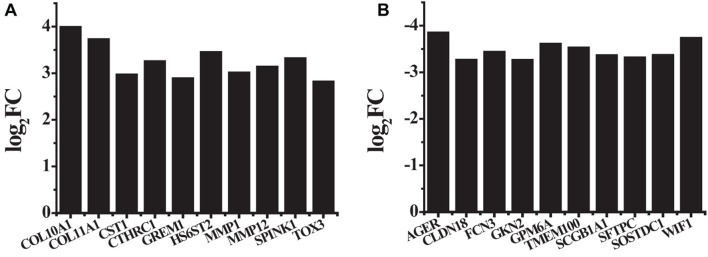
The log_2_FC values of first 10 up-regulated genes and first 10 down-regulated genes that had relatively large differences **(A)** the first 10 up-regulated genes; **(B)** the first 10 down-regulated genes.

A total of 875 differentially expressed genes, including 291 up-regulated genes and 584 down-regulated genes, were obtained with FDR ≤ 0.05 and log 2 FC ≥ 1 criteria. Among these genes, the first 10 up-regulated genes and the first 10 down-regulated genes were shown in Table 2 and Figure 2. The first 10 up-regulated genes were, respectively COL10A1 (log_2_FC = 3.9864, *P* ≤ 0.01), COL11A1 (log_2_FC = 3.7236, *P* ≤ 0.01), CST1 (log_2_FC = 2.9661, *P* ≤ 0.01), CTHRC1 (log_2_FC = 3.2530, *P* ≤ 0.01), GREM1 (log_2_FC = 2.8852, *P* ≤ 0.01), HS6ST2 (log_2_FC = 3.4452, *P* ≤ 0.01), MMP1 (log_2_FC = 3.0086, *P* ≤ 0.01), MMP12 (log_2_FC = 3.1327, *P* ≤ 0.01), SPINK1 (log_2_FC = 3.3138, *P* ≤ 0.01), and TOX3 (log_2_FC = 2.8138, *P* ≤ 0.01), as shown in Figure 2A. The first 10 down-regulated genes were, respectively, AGER (log_2_FC = −3.8451, *P* ≤ 0.01), CLDN18 (log_2_FC = −3.2612, *P* ≤ 0.01), FCN3 (log_2_FC = −3.4334, *P* ≤ 0.01), GKN2 (log_2_FC = −3.2586, *P* ≤ 0.01), GPM6A (log_2_FC = −3.6053, *P* ≤ 0.01), TMEM100 (log_2_FC = −3.5239, *P* ≤ 0.01), SCGB1A1 (log_2_FC = −3.3605, *P* ≤ 0.01), SFTPC (log_2_FC = −3.3127, *P* ≤ 0.01), SOSTDC1 (log_2_FC = −3.3652, *P* ≤ 0.01), and WIF1 (log_2_FC = −3.7317, *P* ≤ 0.01), as shown in Figure 2B.

**TABLE 2:** The log_2_FC values of first 10 up-regulated genes and first 10 down-regulated genes with relatively large differences.

Oncogenes	Names	log_2_FC	*P* value
Up-regulated genes	COL10A1	3.9864	9.69e-32
	COL11A1	3.7236	5.95e-22
	CST1	2.9661	2.90e-23
	CTHRC1	3.2530	9.06e-26
	GREM1	2.8852	2.75e-15
	HS6ST2	3.4452	7.12e-22
	MMP1	3.0086	5.06e-13
	MMP12	3.1327	1.54e-17
	SPINK1	3.3138	1.06e-14
	TOX3	2.8138	2.69e-20
Down-regulated genes	AGER	−3.8451	3.62e-35
	CLDN18	−3.2612	9.98e-19
	FCN3	−3.4334	1.01e-22
	GKN2	−3.2586	3.43e-20
	GPM6A	−3.6053	2.97e-31
	TMEM100	−3.5239	6.07e-21
	SCGB1A1	−3.3605	4.04e-10
	SFTPC	−3.3127	3.23e-15
	SOSTDC1	−3.3652	5.19e-19
	WIF1	−3.7317	2.09e-17

### Up-Regulated Gene Signal Analysis Network

The up-regulated gene COL11A1 was taken as an example; the types of its signal transduction molecules were counted (Figure 2).

**FIGURE 3:**
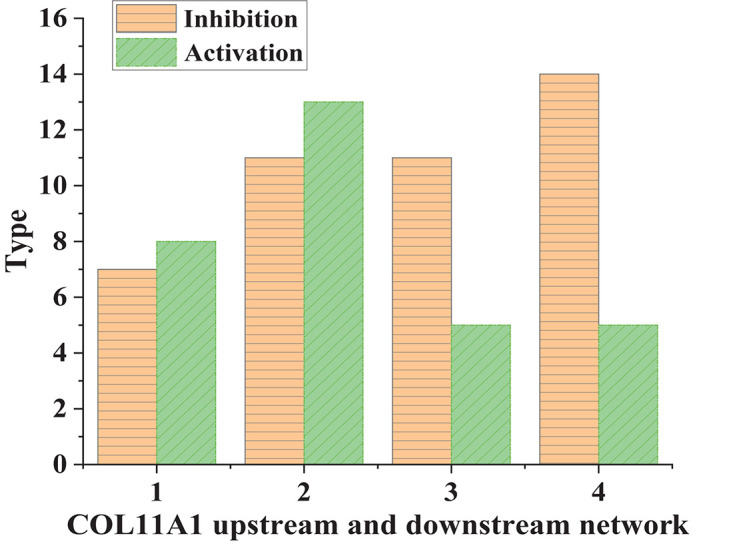
COL11A1 upstream and downstream network signal transduction in different tissues (1 and 3: normal human tissue; 2 and 4: lung cancer tissue).

**FIGURE 4:**
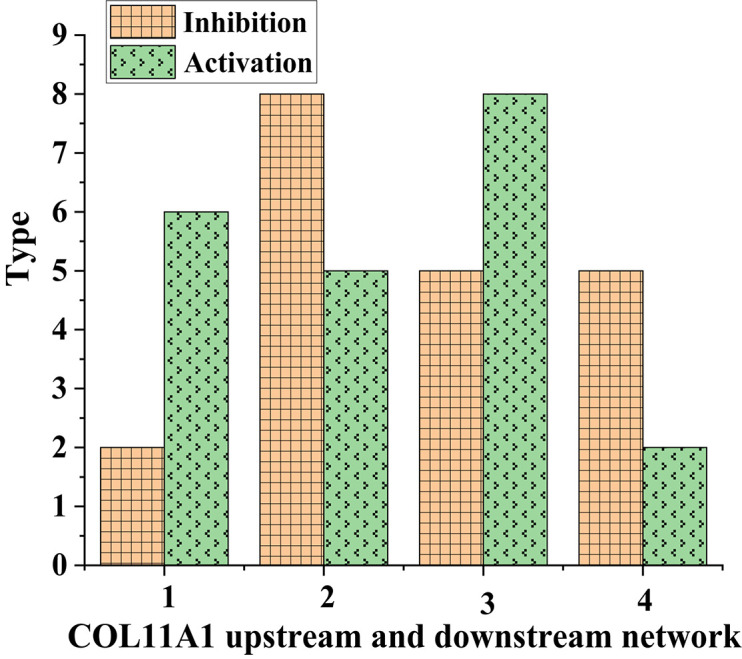
Metastatic molecular types of COL11A1 in different tissues (1 and 3: normal human tissue; 2 and 4: lung cancer tissue).

According to Figure 3, the inhibitory conduction signals in normal human tissues were lower than those in lung cancer tissues. In comparison, the activating conduction signals in lung cancer tissues were generally higher than those in normal tissues. This suggested that COL11A1 was involved in the molecular driving mechanism of lung cancer. Next, the types of transferred molecules of COL11A1 were analyzed (Figure 4).

As shown in Figure 4, the types of metastatic molecules of inhibitory COL11A1 in normal human tissues were lower than those in lung cancer tissues. In contrast, the types of metastatic molecules of activating COL11A1 in lung cancer tissues were more than those in normal tissues. This showed that COL11A1 was very metastatic in lung cancer tissues.

### Signal Pathway Analysis and Biological Function Enrichment Results

**FIGURE 5:**
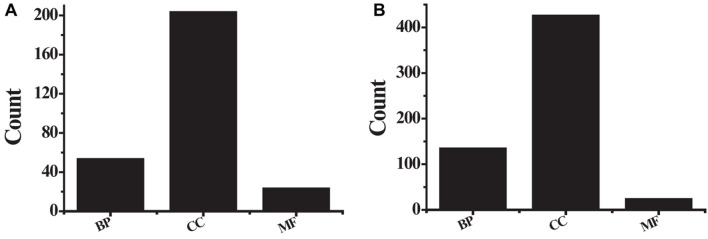
The GO analysis results **(A)** the first 10 up-regulated genes; **(B)** the first 10 down-regulated genes.

The obtained 291 up-regulated genes and 584 down-regulated genes were input into the DAVID platform for signal pathway analysis and biological function enrichment. The results showed that the differentially expressed genes were enriched in 435, with statistically significant differences (*P* ≤ 0.01). The expressed genes were enriched in 166, and the results of GO analysis of the top ten up-regulated genes and the top ten down-regulated genes were shown in Table 3 and Figure 5. Among the up-regulated genes (see Figure 5A), BP contains 53 genes with pathway IDs of GO.0030574, GO.0006508, GO.0030199, and GO.0000281; CC contains pathway IDs of GO.0005615, GO.0005576, GO. 203 genes of 0070062 and GO.0005581; MF contains 23 genes with pathway IDs of GO.0004252 and GO.0004556. Among the down-regulated genes (see Figure 5B), BP contains 134 genes with pathway IDs of GO.0030593, GO.0006954, GO.0006955, GO.0001525, and GO.0050729; CC contains pathway IDs of GO.0005615, GO. 425 genes of 0005576, GO.0005578 and GO.0005886; MF contains 23 genes with pathway ID GO.0008201. For BP, the up-regulated genes mainly occurred in the process of protein metabolism, proteolysis, mitosis, and cell division. The down-regulated genes were mainly reflected in neutrophilic granulocyte chemoattractant, inflammatory reaction, immune response, and angiogenesis. In the process. For CC, up-regulated genes were mainly enriched in extracellular and collagen trimers and down-regulated genes were mainly enriched outside the cell. For MF, the up-regulated genes were mainly expressed in active serine endonuclease and α-amylase, while the down-regulated genes did not show significant enrichment.

**TABLE 3:** The GO analysis results of the first 10 up-regulated genes and first 10 down-regulated genes.

Oncogenes	Gene GO classification	Pathway ID	Pathway description	The quantity of genes	*P* value
Up-regulated gene	BP	GO.0030574	Collagen catabolism	14	1.06e-10
	BP	GO.0006508	Proteolysis	26	8.45e-6
	BP	GO.0030199	Collagen fiber tissue	7	1.62e-6
	BP	GO.0000281	Mitosis and cell division	6	4.63e-5
	CC	GO.0005615	Extracellular	53	9.56e-11
	CC	GO.0005576	Extracellular	55	7.45e-9
	CC	GO.0070062	Extracellular	83	1.56e-7
	CC	GO.0005581	Collagen trimer	12	9.63e-7
	MF	GO.0004252	Active serine endonuclease activity	19	1.45e-8
	MF	GO.0004556	α-amylase	4	2.74e-7
Down-regulated gene	BP	GO.0030593	Neutrophilic granulocyte chemoattractant	19	1.07e-13
	BP	GO.0006954	Inflammatory response	38	3.14e-11
	BP	GO.0006955	Immunological reaction	37	1.01e-11
	BP	GO.0001525	Angiogenesis	26	9.45e-8
	BP	GO.0050729	Inflammatory reaction	14	4.06e-8
	CC	GO.0005615	Extracellular	106	9.88e-17
	CC	GO.0005576	Extracellular	108	1.04e-18
	CC	GO.0005578	Extracellular matrix	33	1.16e-13
	CC	GO.0005886	Cytoplasm membrane	178	6.05e-8
	MF	GO.0008201	Heparin binding	23	4.33e-8

**FIGURE 6:**
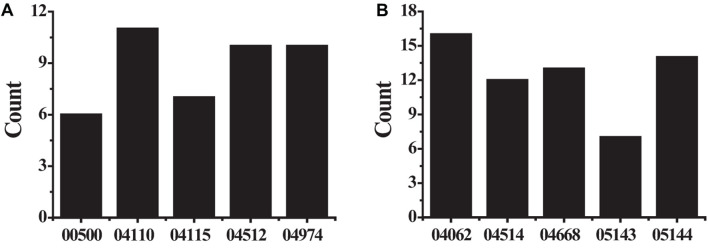
The KEGG pathway analysis results **(A)** showed the differently expressed up-regulated genes; **(B)** showed the differently expressed down-regulated genes.

The KEGG pathway analyzed the biological functions of genes from the system level through abundant pathway information, including many complex biological functions such as genetic information transmission, metabolic pathways, and cellular processes. From the annotation analysis of a single gene to the annotation analysis of a gene set, it is judged whether a group of genes appears on a functional node. The KEGG pathway analysis identifies biological processes most relevant to biological phenomena and greatly enhances the reliability of the survey. The results of the KEGG pathway analysis of differentially expressed up-regulated genes and differentially expressed down-regulated genes were shown in Table 4. As shown in Figure 6, the up-regulated genes include six genes (see Figure 6A) with pathway ID 00500: AMY1A, AMY1B, AMY1C, AMY2A, AMY2B, and PGM2L1; 11 genes of 04110: BUB1B, CCNB1, CDC20, CDK1, CDKN2A, MAD2L1, MCM2, ORC6, PTTG1, SFN, and TTK; 7 genes with pathway ID 04115: CCNB1, CDK1, CDKN2A, IGFBP3, RRM2, SFN, and STEAP3 10 genes with pathway ID: 04512: COL1A1, COL1A2, COL3A1, COL5A1, COL5A2, COL11A1, COMP, HMMR, SPP1, and THBS2; 10 genes with pathway ID 04974: ACE2, COL1A1, COL1A2, COL3A1, COL5A1, COL5A2, COL11A1, DPP4, KCNN4, and KCNK5. Down-regulated genes include 16 genes (see Figure 6B) with pathway ID 04062: ARRB1, CCL2, CCL4, CCL14, CCL21, CCL23, CXCL3, CXCL12, CXCR2, CX3CL1, ELMO1, FGR, GNG11, PLCB4, PPBP, and PREX1; pathway ID is 04514 Genes: CADM1, CDH5, CD274, CLDN5, CLDN18, CLDN22, ESAM, ICAM1, ICAM2, PECAM1, PTPRM, and SELP; 13 genes with pathway ID 04668: CCL2, CXCL3, CX3CL1, EDN1, FOS, ICAM1, IL1B, IL18R1, IL6, JUNB, MAP3K8, PTGS2, and TNFAIP3; 7 genes with pathway ID 05143: ICAM1, IL1B, IL6, HBA1, HBA2, HBB, and PLCB4; 14 genes with pathway ID 05144: CCL2, CD36, CSF3, GYPC, HBA1, HBA2, HBB, ICAM1, IL1B, IL6, KLRB1, PECAM1, SELE, and SELP. The up-regulated genes were mainly enriched in cell cycle, extracellular matrix receptor interaction, protein digestion and absorption, p53 signaling pathway, starch and sucrose metabolism, and down-regulated genes were mainly enriched in chemokine signal transduction pathways, malaria, TNF (tumor necrosis factor) signaling pathway, cell adhesion molecules, African trypanosomiasis pathway.

**TABLE 4:** The KEGG pathway analysis results of differently expressed up-regulated genes and differently expressed down-regulated genes.

Oncogenes	Pathway ID	Amount of genes	Names	Pathway description	*P* value
Up-regulated genes	00500	6	AMY1A, AMY1B, AMY1C, AMY2A, AMY2B, PGM2L1	Starch and sucrose metabolism	2.34e-4
	04110	11	BUB1B, CCNB1, CDC20, CDK1, CDKN2A, MAD2L1, MCM2, ORC6, PTTG1, SFN, TTK	Cell cycle	1.06e-5
	04115	7	CCNB1, CDK1, CDKN2A, IGFBP3, RRM2, SFN, STEAP3	p53 signaling pathway	9.95e-4
	04512	10	COL1A1, COL1A2, COL3A1, COL5A1, COL5A2, COL11A1, COMP, HMMR, SPP1, THBS2	Extracellular matrix receptor interaction	2.01e-5
	04974	10	ACE2, COL1A1, COL1A2, COL3A1, COL5A1, COL5A2, COL11A1, DPP4, KCNN4, KCNK5	Digestion and absorption of protein	2.78e-6
Down-regulated genes	04062	16	ARRB1, CCL2, CCL4, CCL14, CCL21, CCL23, CXCL3, CXCL12, CXCR2, CX3CL1, ELMO1, FGR, GNG11, PLCB4, PPBP, PREX1	Chemokine signaling transduction pathway	1.37e-5
	04514	12	CADM1, CDH5, CD274, CLDN5, CLDN18, CLDN22, ESAM, ICAM1, ICAM2, PECAM1, PTPRM, SELP	Cell adhesion molecule	1.46e-3
	04668	13	CCL2, CXCL3, CX3CL1, EDN1, FOS, ICAM1, IL1B, IL18R1, IL6, JUNB, MAP3K8, PTGS2, TNFAIP3	TNF signaling pathway	1.69e-6
	05143	7	ICAM1, IL1B, IL6, HBA1, HBA2, HBB, PLCB4	African trypanosomiasis	1.54e-4
	05144	14	CCL2, CD36, CSF3, GYPC, HBA1, HBA2, HBB, ICAM1, IL1B, IL6, KLRB1, PECAM1, SELE, SELP	Malaria	1.01e-11

### PPI Analysis Results

**FIGURE 7:**
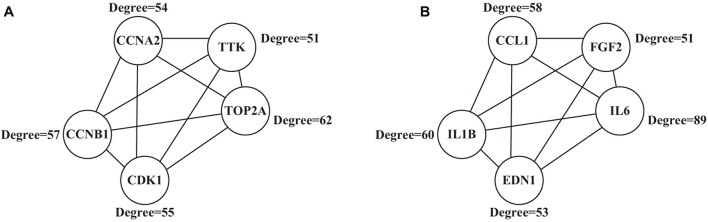
Significant modules in the protein-protein interaction network **(A)** showed the highly expressed genes; **(B)** showed the low expressed genes.

A 292 nodes and 1,425 interaction networks were obtained from 291 up-regulated genes, and 529 nodes and 1,624 interaction networks were obtained from 584 down-regulated genes by analyzing the string tool. After processing with visualization software, the significant module in the protein-protein interaction relationship network in Figure 7 was obtained, and the high expression in the center of the protein-protein interaction network was selected from the protein-protein interaction network. The gene (Figure 7A), included TOP2A (Degree = 62), CCNB1 (Degree = 57), CCNA2 (Degree = 54), CDK1 (Degree = 55), and TTK (Degree = 51), all of which have larger mutual Acting relationship. A low-expression gene at the center of the protein-protein interaction network (Figure 7B), including IL6 (Degree = 89), IL1B (Degree = 60), CCL1 (Degree = 58), EDN1 (Degree = 53), and FGF2 (Degree = 51) had a large interaction relationship. These highly expressed genes and low expressed genes may be key target genes related to lung cancer diseases.

### Protein Expressions of High-Expressed and Low-Expressed Genes

**FIGURE 8:**
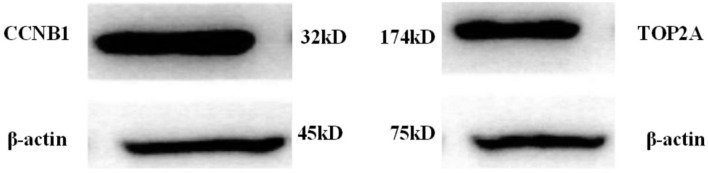
Protein expressions of CCNB1 and TOP2A in lung cancer patients.

**FIGURE 9:**
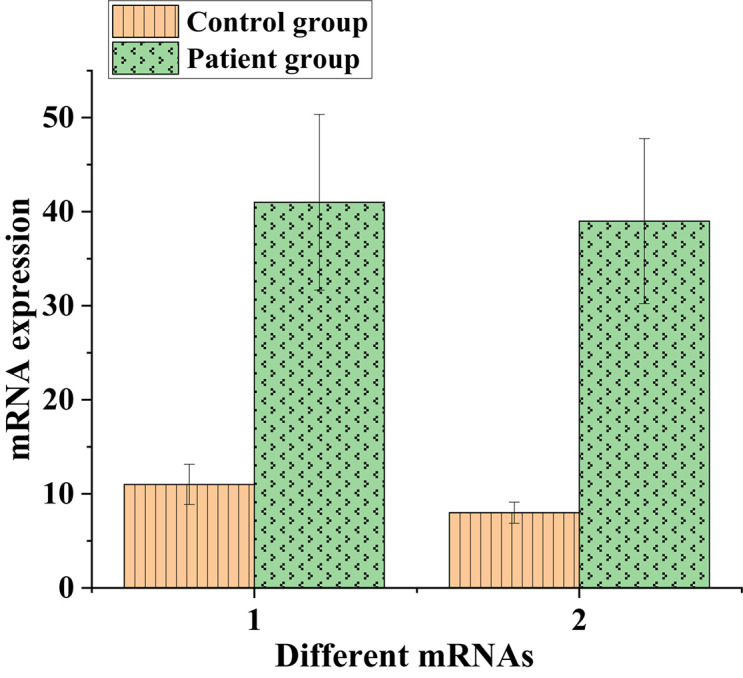
The expression of messenger RNA corresponding to CCNB1 and TOP2A proteins in the control and experimental groups (1: CCNB1; 2: TOP2A).

Protein expressions of the high-expressed genes CCNB1 and TOP2A were illustrated in Figure 8 below:

Afterward, the expression of messenger RNA corresponding to CCNB1 and TOP2A proteins was analyzed, and the results were shown in Figure 9 below.

As shown in Figures 8, 9, CCNB1 and TOP2A proteins corresponding to the messenger RNA expression level in normal humans were around 10, while the CCNB1 and TOP2A protein corresponding to the messenger RNA expression level in the patient group both exceeded 35, indicating that CCNB1 and TOP2A proteins were highly expressed in patients.

**FIGURE 10:**
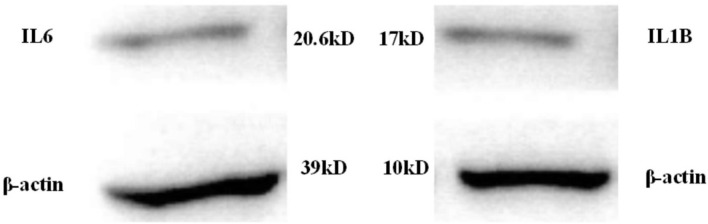
Protein expressions of IL6 and IL1B in patients with lung cancer.

**FIGURE 11:**
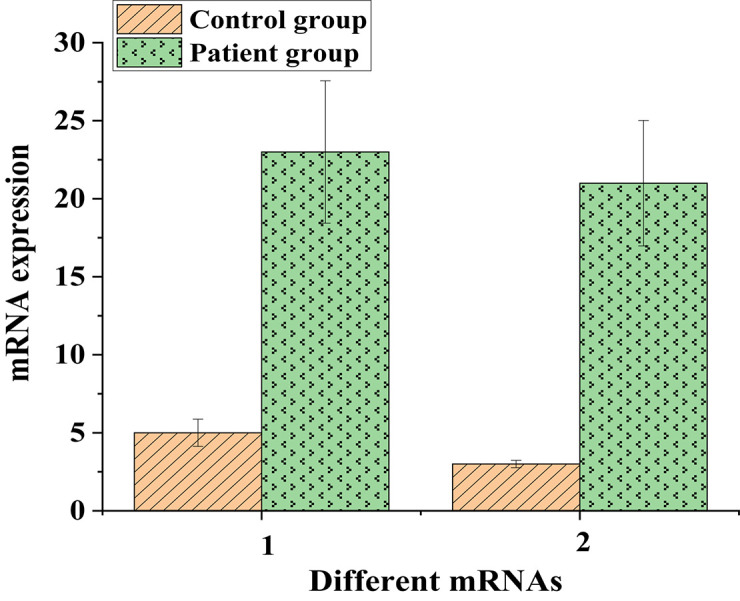
The messenger RNA expressions of IL6 and IL1B proteins in the control and experimental groups (1: IL6; 2: IL1B).

Protein expressions of the low-expressed genes IL6 and IL1B were illustrated in Figure 10 below:

Then, the messenger RNA expression of IL6 and IL1B proteins was analyzed (Figure 11).

As shown in Figures 10, 11, protein expressions of the low-expressed genes IL6 and IL1B in patients were low. The messenger RNA expressions corresponding to IL6 and IL1B proteins in the control group were around 5, while they both exceeded 25 in the patient group. This suggested that even though IL6 and IL1B proteins were low-expressed in patients, they were still lung cancer-related target genes.

## Conclusion

This study attempts to reveal the molecular driving mechanism of lung cancer through signal pathway, biological function enrichment, protein interaction analysis, and gene target network analysis. A total of 875 differentially expressed genes were obtained by analyzing the samples. These genes are mainly involved in biological processes such as protein metabolism, protein hydrolysis, mitosis and cell division. TOP2A, CCNB1, CCNA2, CDK1, and TTK may be the key target genes of lung cancer. Exploring the changes of various genes and pathways in the pathogenesis of lung cancer provides reference for the molecular driving mechanism of lung cancer, and provide theoretical basis for molecular-targeted drug therapy and clinical nursing guidance of lung cancer. However, there are still some shortcomings. The selection number of up-regulated and down-regulated genes is limited, which cannot meet the huge molecular network analysis. In the later stage, the screening amount of up-regulated and down-regulated genes will be increased. The molecular driving mechanism of lung cancer was still in the preliminary stage. In the subsequent research, TOP2A with large interaction relations among the critical target genes related to lung cancer obtained by screening would be screened for drug resistance, providing assistance for the development of its inhibitors.

## Data Availability Statement

Publicly available datasets were analyzed in this study. This data can be found here: the GEO (Gene Expression Omnibus) database.

## Author Contributions

RH: writing – original draft and conceptualization. XX: data curation and software. KZ: supervision and resources. YZ: formal analysis. CW: validation. GH: writing, review, editing, and methodology. All authors contributed to the article and approved the submitted version.

## Conflict of Interest

The authors declare that the research was conducted in the absence of any commercial or financial relationships that could be construed as a potential conflict of interest.

## Publisher’s Note

All claims expressed in this article are solely those of the authors and do not necessarily represent those of their affiliated organizations, or those of the publisher, the editors and the reviewers. Any product that may be evaluated in this article, or claim that may be made by its manufacturer, is not guaranteed or endorsed by the publisher.

## References

[B1] Allan-Blitz L. T. Hemarajata P. Humphries R. M. Wynn A. Segura E. R. Klausner J. D. A. (2018). Cost analysis of gyrase a testing and targeted ciprofloxacin therapy versus recommended 2-drug therapy for *Neisseria gonorrhoeae* infection. *Sex. Transm. Dis.* 45 87–91. 10.1097/olq.0000000000000698 29329176 PMC6879096

[B2] Horimasu Y. Ishikawa N. Taniwaki M. Yamaguchi K. Hamai K. Iwamoto H. (2017). Gene expression profiling of idiopathic interstitial pneumonias (IIPs): identification of potential diagnostic markers and therapeutic targets. *BMC Med. Genet.* 18:88. 10.1186/s12881-017-0449-9 28821283 PMC5562997

[B3] Krasnov G. S. Puzanov G. A. Kudryavtseva A. V. Dmitriev A. A. Beniaminov A. D. Kondratieva T. T. (2017). Differential expression of an ensemble of the key genes involved in cell-cycle regulation in lung cancer. *Mol. Biol.* 51 740–747. 10.1134/s0026893317050107 29116073

[B4] Masters G. A. Johnson D. H. Temin S. (2017). Systemic therapy for stage IV non-small-cell lung cancer: american society of clinical oncology clinical practice guideline update. *J. Oncol. Pract.* 33 832–837. 10.1200/jop.2017.026716 28850309

[B5] Shen J. Hu Y. Putt K. S. Singhal S. Han H. Visscher D. W. (2018). Assessment of folate receptor alpha and beta expression in selection of lung and pancreatic cancer patients for receptor targeted therapies. *Oncotarget* 9 4485–4495. 10.18632/oncotarget.23321 29435118 PMC5796989

[B6] Shi Y. Sun Y. Yu J. Ding C. Wang Z. Wang C. (2017). China experts consensus on the diagnosis and treatment of advanced stage primary lung cancer (2016 version). *Zhongguo Fei Ai Za Zhi* 19 1–15. 10.21147/j.issn.1000-9604.2019.01.01 26805732 PMC5999802

[B7] Wang L. Qu J. Liang Y. Zhao D. Rehman F. U. Qin K. (2020). Identification and validation of key genes with prognostic value in non-small-cell lung cancer via integrated bioinformatics analysis. *Thorac. Cancer* 11 851–866. 10.1111/1759-7714.13298 32059076 PMC7113067

[B8] Xu R. Xu R. Zhong G. Huang T. He W. Kong C. (2018). Sequencing of circulating tumor DNA for dynamic monitoring of gene mutations in advanced non-small cell lung cancer. *Oncol. Lett.* 15 3726–3734. 29556275 10.3892/ol.2018.7808 PMC5843997

[B9] Zhang Y. Luo J. Wang X. Wang H. L. Zhang X. L. Gan T. Q. (2018). A comprehensive analysis of the predicted targets of miR-642b-3p associated with the long non-coding RNA HOXA11-AS in NSCLC cells. *Oncol. Lett.* 15 6147–6160. 29616096 10.3892/ol.2018.8105 PMC5876445

[B10] Zhou G. Ye J. Fang Y. Zhang Z. Zhang J. Sun L. (2017). Identification of DBCCR1 as a suppressor in the development of lung cancer that is associated with increased DNA methyltransferase 1. *Oncotarget* 8 32821–32832. 10.18632/oncotarget.15826 28427182 PMC5464830

[B11] Zins K. Heller G. Mayerhofer M. Schreiber M. Abraham D. (2018). Differential prognostic impact of interleukin-34 mRNA expression and infiltrating immune cell composition in intrinsic breast cancer subtypes. *Oncotarget* 9 23126–23148. 10.18632/oncotarget.25226 29796177 PMC5955405

